# Development and Validation of In‐House Conventional and Multiplex PCR Methods for the Detection and Identification of *Lophomonas* spp.: An Innovative Approach

**DOI:** 10.1002/jcla.70049

**Published:** 2025-05-08

**Authors:** Maryam Nakhaei, Mahdi Fakhar, Abouzar Bagheri, Hajar Ziaei Hezarjaribi, Saied Abediankenari, Ali Sharifpour, Maryam Ghasemi

**Affiliations:** ^1^ Toxoplasmosis Research Center Communicable Diseases Institute, Mazandaran University of Medical Sciences Sari Iran; ^2^ Iranian National Registry Center for Lophomoniasis (INRCL) Imam Khomeini Hospital, Mazandaran University of Medical Sciences Sari Iran; ^3^ Department of Medical Microbiology and Immunology School of Medicine, Qom University of Medical Sciences Qom Iran; ^4^ Molecular and Cell Biology Research Center, Faculty of Medicine Mazandaran University of Medical Sciences Sari Iran; ^5^ Immunogenetics Research Centre, Faculty of Medicine Mazandaran University of Medical Sciences Sari Iran; ^6^ Department of Pathology, Faculty of Medicine Mazandaran University of Medical Sciences Sari Iran

**Keywords:** conventional PCR, diagnosis, Iran, *Lophomonas*, microscopic examination, multiplex PCR

## Abstract

**Background:**

Pulmonary lophomoniasis is an emerging disease caused by the protozoan pathogen *Lophomonas* spp. Recently, a conventional polymerase chain reaction (PCR) method has been developed. However, its sensitivity and specificity remain to be fully established. Therefore, this study aimed to develop in‐house conventional and multiplex PCR for the detection and identification of *Lophomonas* infections. Additionally, we attempted to compare the diagnostic performance of these novel PCR tests with the current microscopic examination method using BAL samples.

**Methods:**

We studied 120 bronchoalveolar lavage (BAL) specimens of the patients clinically suspected of having lophomoniasis. The specimens were examined using three methods: microscopic examination (Giemsa staining), in‐house conventional PCR, and multiplex‐PCR. Moreover, multiplex‐PCR was used for the simultaneous identification of two species of *Lophomonas*.

**Results:**

Out of the 120 BAL specimens tested, 30 (25%) tested positive through microscopic wet mount examination. Among the three techniques, multiplex‐PCR was the most sensitive (100%, 95% CI, 88.3–100), while Giemsa staining had the lowest sensitivity (86.2%, 95% CI, 69.4–94.5). The data reveal a strong agreement between multiplex‐PCR and conventional PCR (κ = 0.96), while the lowest agreement was found between multiplex‐PCR and microscopy methods (κ = 0.16). The study also confirmed the presence of *L. blattarum* species in all samples using multiplex‐PCR.

**Conclusions:**

This study demonstrates that the in‐house multiplex‐PCR is a robust and accurate diagnostic test for the detection and identification of *Lophomonas* species. Therefore, our findings suggest that this method may be a powerful tool to overcome some diagnostic pitfalls for lophomoniasis.

## Graphical abstract

Sequence alignment of the SSU rRNA of the two *Lophomonas* species, *L. blattarum* and *L. striata* . The red rectangle highlights the position of the reverse universal primer; the yellow, the *L. blattarum*‐specific forward primer; and the green, the *L. striata* ‐specific forward primer used in multiplex PCR. The 3′ end of each primer was designed based on targeting nucleotides distinguishing the two species.

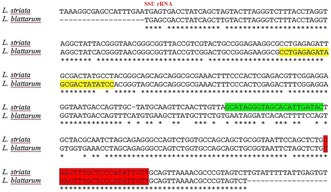

## 1 Introduction

*Lophomonas* spp. is a neglected protozoan belonging to the order Hypermastigida and suborder Lophomonadina. Two species of *Lophomonas*, *L. blattarum* and *L. striata* , have been identified to date. This emerging protozoan acts as a facultative commensal agent in the hindgut of termites and cockroaches [1, 2]; however, the epidemiological, clinical, and diagnostic aspects of this parasite are not well understood [3, 4]. *Lophomonas* exists in two forms: the trophozoite and the cyst. The pleomorphic trophozoite is typically 20–60 μm long and 10–20 μm wide, with motile gangs of flagella. The cyst is spherical‐elliptical and encased in a homogeneous membrane. When inhaled, cysts can infect the respiratory tract of patients, leading to infections in various organs such as the sinuses, lungs, and other parts of the respiratory tract. The clinical manifestations of respiratory tract infections caused by *Lophomonas* have similarities with those caused by other common pathogens including fungi, viruses, and bacteria [5, 6].

Since the first report of human infection with *Lophomonas* in China in 1993, this disease has been documented in 10 countries across four continents, predominantly in Asia [7]. The global prevalence of lophomoniasis is not well established, but estimates suggest it is approximately 22% in Iran [3, 4]. The primary clinical symptoms reported by patients include chronic cough, expectoration, dyspnea, and, in some cases, hemoptysis [4, 5, 6].

Lophomoniasis can affect patients ranging from 1 month to 84 years old, and the drug treatment of choice is metronidazole. For diagnosis, bronchoalveolar lavage (BAL) and sputum are commonly used, and microscopic examination is typically used to identify the multi‐flagellated protozoan. However, identifying *Lophomonas* can be challenging as it closely resembles respiratory epithelial cells in morphology [4, 7]. On the other hand, the absence of a standardized culture medium and serological testing for this emerging parasite has encouraged researchers to shift their focus towards more advanced methods, such as molecular approaches. To address this issue, researchers have proposed a molecular diagnostic technique to distinguish the parasite from bronchial and nasal epithelial cells. In 2019, the Iranian National Registry Center for Lophomoniasis (INRCL) research group developed the first conventional genus‐specific polymerase chain reaction (PCR) test for diagnosing lophomoniasis [8]. All previous molecular‐based studies conducted for the detection of *Lophomonas* spp., using BAL and/or sputum samples, have been developed by our research group at INRCL, utilizing genus‐specific PCR tests [9, 10, 11, 12]. These studies highlight the effectiveness of PCR in detecting *Lophomonas*. However, the sensitivity and specificity of this diagnostic test have not yet been fully determined. Furthermore, differentiation between the two *Lophomonas* species has not been established to date. Thus, we developed in‐house conventional and multiplex PCR assays for detecting and identifying *Lophomonas* infections. Furthermore, we compared the diagnostic performance of these novel PCR tests with the current microscopic examination method using BAL samples. The performance metrics assessed included sensitivity, specificity, positive predictive value (PPV), negative predictive value (NPV), likelihood ratio, and overall accuracy.

## 2 Patients and Methods

### 2.1 Sample Collection

Between December 2020 and July 2021, a total of 120 BAL specimens were collected from patients clinically suspected of having lophomoniasis. These patients exhibited symptoms such as chronic cough, expectoration, dyspnea, hemoptysis, and showed either poor or no response to current therapy. The specimens were sent to the INRCL at Mazandaran University of Medical Sciences (MAZUMS) in Sari, northern Iran, for further evaluation to confirm or rule out the presence of this disease. Out of all BAL submitted specimens, 30 (30/120; 25%) were confirmed to have lophomoniasis through direct microscopic examination (wet mount), and they responded well to the prescribed therapy. To compare the diagnostic performance of three different methods for diagnosing lophomoniasis in these confirmed patients, routine microscopic observation of BAL specimens was used alongside newly developed in‐house conventional PCR (genus‐specific) and multiplex‐PCR methods (species‐specific). To assess sensitivity, we analyzed 30 BAL samples from confirmed lophomoniasis cases using three diagnostic tests. Additionally, to evaluate the specificity of these diagnostic methods, we collected 30 BAL specimens from patients who tested negative for *Lophomonas* infection but were diagnosed with other respiratory conditions including lung tuberculosis (*n* = 7), COPD (*n* = 6), Asthma (*n* = 5), pneumocystis (*n* = 3), lung aspergillosis (*n* = 2), lung hydatid cyst (*n* = 3), and COVID‐19 (*n* = 4).

### 2.2 Microscopic Examination (Wet Mount and Giemsa Staining Methods)

In total, 30 BAL specimens were subjected to direct microscopy examination using Giemsa staining. Prior to examination, the specimens were centrifuged for 2 min at 500 *g* to concentrate the material for better visualization. A wet mount procedure was performed by placing a drop of the sediment on a microscope slide and covering it with a coverslip. In cases where the specimen is transparent and contains purulent mucus, centrifugation is not necessary. A small sample of the purulent material is then taken, placed on a slide, and covered with a coverslip for microscopic examination. The prepared slides were examined under a light microscope at a magnification of 400× to observe the motile and live pleomorphic trophozoites of *Lophomonas* spp., along with their variations of shape and size, and predominantly aggregated clumps [4]. The microscopic examination was performed by a skilled laboratory technician who was blinded to the results of the other diagnostic tests. The presence of multiflagellates and their characteristic morphology were used as the diagnostic criteria for *Lophomonas* infection. Next, two smears were prepared from each BAL specimen. These smears should ideally be relatively thick and not scattered, resembling the circular shape of a thick blood film used in malaria diagnosis, to increase the chances of detection. The smears were then allowed to dry and fixed with methanol. Following fixation, the slides were flooded with 5% buffered Giemsa solution (pH 7.4) for 45 min. The slides were then allowed to dry at room temperature and examined under light microscopy at magnifications of 400× and 1000× to facilitate the identification of *Lophomonas* spp. The results were recorded and compared with the findings of the conventional PCR and multiplex‐PCR methods.

### 2.3 DNA Extraction

For DNA extraction, a total of 200 μL of each BAL specimen was homogenized in 200 μL of digestive buffer consisting of 50 mM Tris–HCl (pH 7.6), 1 mM EDTA, and 1% Tween 20. Subsequently, 20 μL of proteinase K solution containing 20 mg/mL of enzyme was added to the mixture, which was then incubated overnight at 45°C. Next, the homogenate was treated with 200 μL of phenol, chloroform, and isoamyl alcohol (25:24:1) solution and thoroughly mixed by shaking. After centrifugation at 14,000 *g* for 15 min, the supernatant was carefully transferred into a new microtube, to which 400 μL of cold 100% ethanol was added, followed by storage at −20°C for 2 h. The sediment was then treated with 200 μL of 70% ethanol, centrifuged, and resuspended in 50 μL of double‐distilled water, which was kept at 4°C until further use.

### 2.4 In‐House Conventional PCR (Genus‐Specific rRNA PCR)

A pair of primers was developed for amplifying the genus‐specific rRNA of *Lophomonas* spp. This was achieved by aligning the small subunit ribosomal ribonucleic acid (SSU rRNA) sequences of two *Lophomonas* species, *L. blattarum* and *L. striata* (GenBank accessions: JX020505.1 and JN088049.1, respectively), through Clustal W [13]. The forward and reverse primers were created with the assistance of OLIGO 7 primer analysis software. The amplification reaction was carried out in a total volume of 25 μL, consisting of 12.5 μL of Master Mix (Fermentas Inc.), 1 μL of each forward (5′‐GAG AAG GCG CCT GAG AGA T‐3′) and reverse (5′‐GCT AGG TTT CAC CAC ACT GGA‐3′) primers. The reaction mixture was subjected to 35 cycles of amplification using a thermocycler (Corbett Research, Sydney, Australia). The cycling conditions consisted of an initial denaturation step at 94°C for 2 min, followed by 40 cycles of denaturation at 94°C for 1 min, annealing at 57°C for 1 min, and extension at 72°C for 1 min, with a final extension step at 72°C for 3 min. The amplified products (6 μL) were electrophoresed on a 2% (*w*/*v*) agarose gel in Tris–Borate–EDTA (TBE) buffer. The gel was stained with SYBR Safe Stain (Invitrogen), and the amplified products were visualized using UV transillumination. Positive and negative controls were included in all PCR tests, consisting of *Lophomonas* DNA from previous work [9] and water without DNA, respectively. The PCR product was 211 bp for *Lophomonas* genus.

### 2.5 In‐House Multiplex‐ PCR (Species‐Specific rRNA PCR)

**FIGURE 1:**
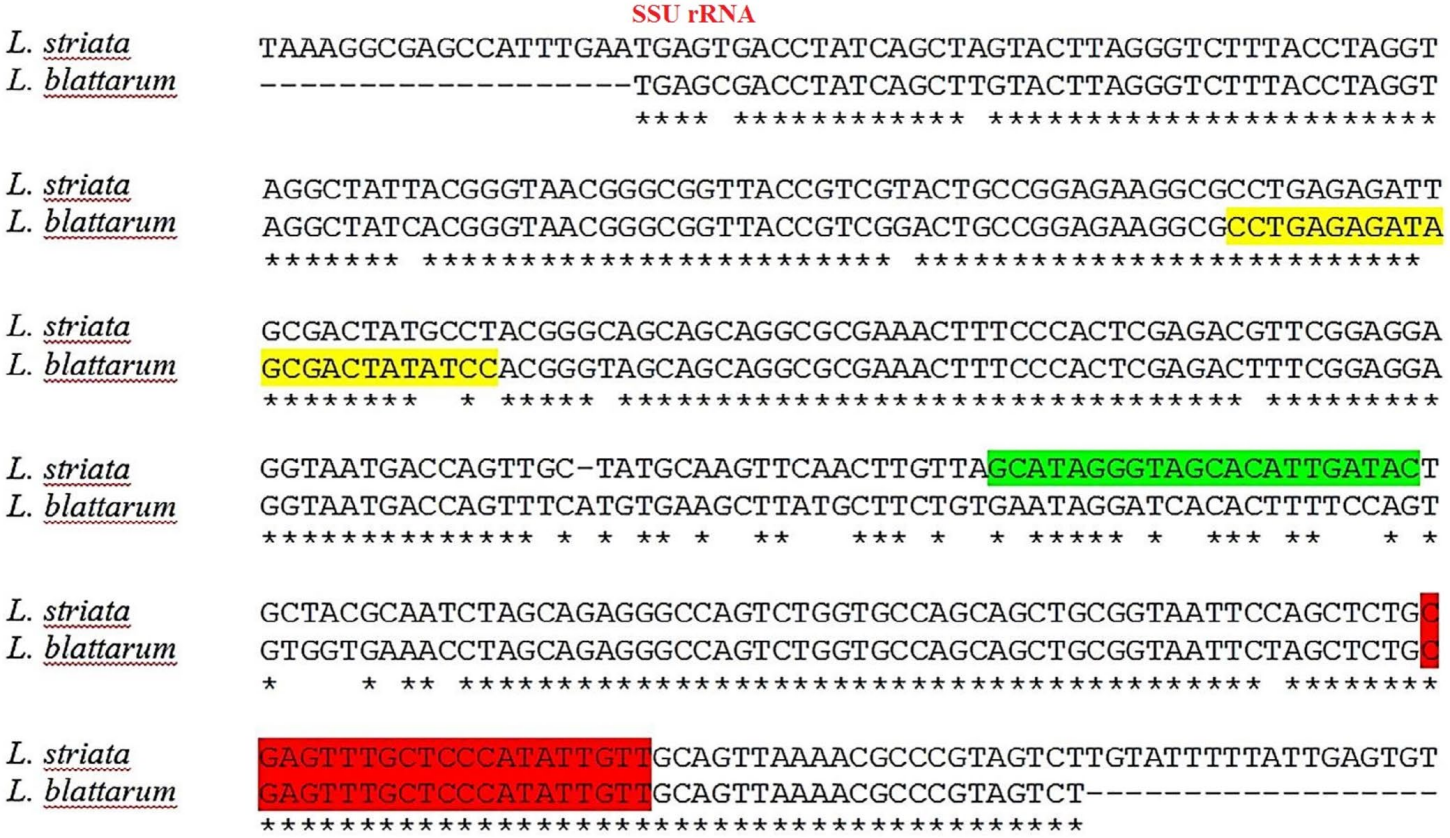
Sequence alignment of the SSU rRNA of the tow *Lophomonas* species, *L. blattarum* and *L. striata* . The red rectangle highlights the position of the reverse universal primer; the yellow, the *L. blattarum*‐specific forward primer; and the green, the *L. striata* ‐specific forward primer used in multiplex PCR. The 3′ end of each primer was designed based on targeting nucleotides distinguishing the two species.

We designed the three primers for the amplification of species‐specific rRNA of *Lophomonas* species, by aligning the SSU rRNA sequence of two *Lophomonas* species: *L*. *blattarum* and *L. striata* (GenBank accessions: JX020505.1 and JN088049.1, respectively) using Clustal W [13]. (See Figure 1). The forward and reverse primers were designed using OLIGO 7 primer analysis software. The PCR was set up as follows in a total volume of 40 μL, consisting of 20 μL of the Master Mix (Fermentas Inc.), 1.6 μL of each forward (F) primer specific to *L. blattarum* (5′‐CCT GAG AGA TAG CGA CTA TAT CC‐3′) and *L. striata* (5′‐GCA TAG GGT AGC ACA TTG ATA C‐3′), 1.6 μL of the reverse (R) primer (5′‐AAC AAT ATG GGA GCA AAC TCG‐3′), 8 μL of the extracted DNA, and 7.2 μL of distilled water. The primers were designed from SSU rRNA. The PCR amplification was performed in the same way as the genus‐specific rRNA PCR, with 35 cycles at 94°C for 1 min, 57°C for 1 min, and 72°C for 1 min. The resulting multiplex PCR products were 211 bp and 103 bp for *L. blattarum* and *L. striata* , respectively.

To serve as positive controls, *L. blattarum* DNA was extracted from specimens that were previously registered in the gene bank (NCBI). However, due to a lack of access to *L. striata* DNA, the sequence of the *L. striata* genome fragment was extracted from the NCBI database and synthesized by cloning into the pUC57‐T vector (Shinegene, China). The sequence of the synthesized genome fragment is as follows: (5′‐GCA TAG GGT AGC ACA TTG ATA CTG CTA CGC AAT CTA GCA GAG GGC CAG TCT GGT GCC AGC AGC TGC GGT AAT TCC AGC TCT GCG AGT TTG CTC CCA TAT TGT T‐3′).

### 2.6 Data Analysis

The agreement level was assessed using SPSS version 20.0 by calculating Kappa (κ) values with 95% confidence intervals (SPSS Inc., Chicago, IL). Differences between groups were determined using the *χ* ^2^ test, and statistical significance was established at a *p* < 0.05. Kappa values indicate the level of agreement beyond chance, where a value ranging from 0.21 to 0.60 denotes fair to moderate agreement, 0.60–0.80 denotes strong agreement, and ≥ 0.81 indicates almost perfect agreement [14]. Statistical analyses were performed using MedCalc software to determine sensitivity, specificity, PPV, NPV, accuracy, and likelihood ratio estimates. In essence, diagnostic accuracy provides an overall assessment of how well a diagnostic test distinguishes between individuals who have a certain condition and those who do not. A high diagnostic accuracy indicates that the test is reliable and effective in correctly diagnosing the presence or absence of the condition in question. Receiver Operating Characteristic (ROC) curve analysis was also conducted to assess the diagnostic accuracy of each test, allowing for optimal cut‐off values to be established. Additionally, the composite reference standard for each diagnostic test was based on two out of three positive findings obtained from individual research areas, which included microscopy, conventional PCR, or multiplex PCR.

## 3 Results

**FIGURE 2:**
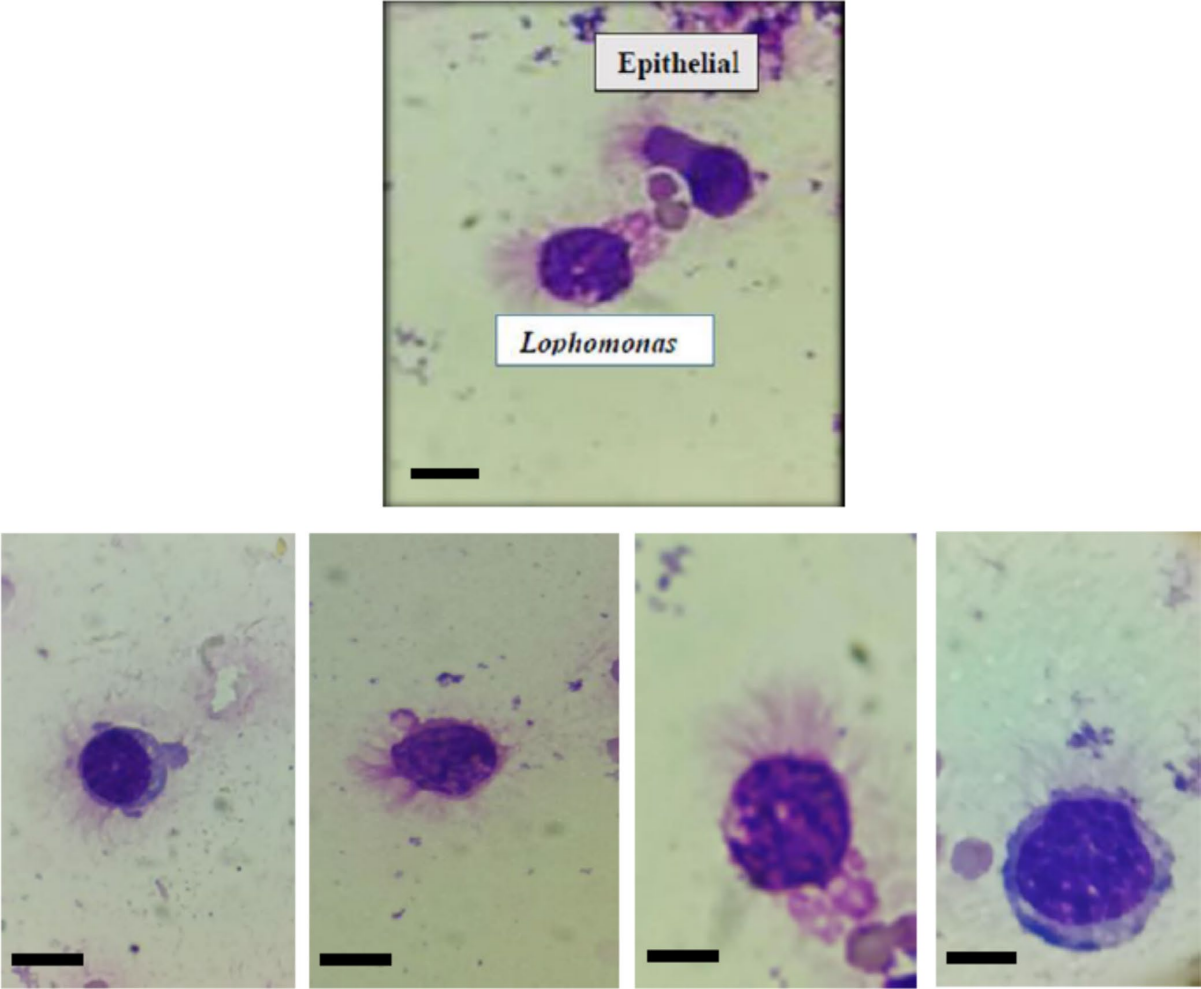
Photomicrograph showing multiple pleomorphic trophozoites of *Lophomonas* from different patients, stained with Giemsa (×1000); Scale bar = 10 nm.

**FIGURE 3:**
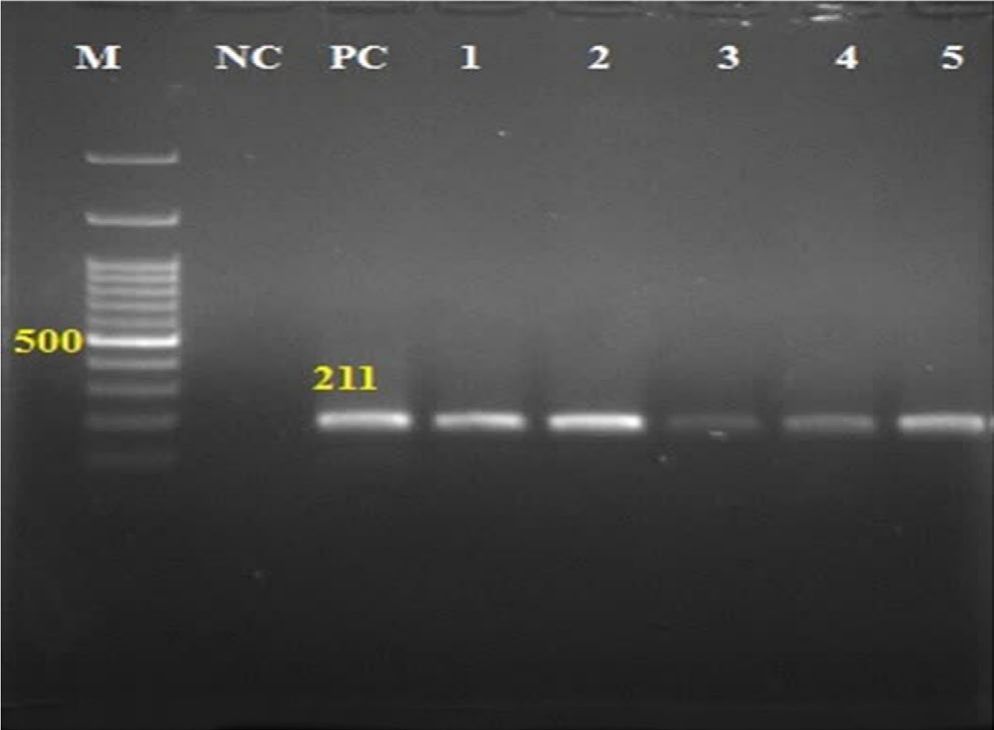
A 211 bp band from the PCR products of positive BAL samples infected with *Lophomonas* spp. by conventional amplification technique in 2% agarose gel electrophoresis. M = marker (100 bp); NC = negative control (distilled H_2_O); PC = positive control (*L. blattarum* DNA; accession numbers: MN243135); 1–5 = positive specimens (patient's BAL samples).

**FIGURE 4:**
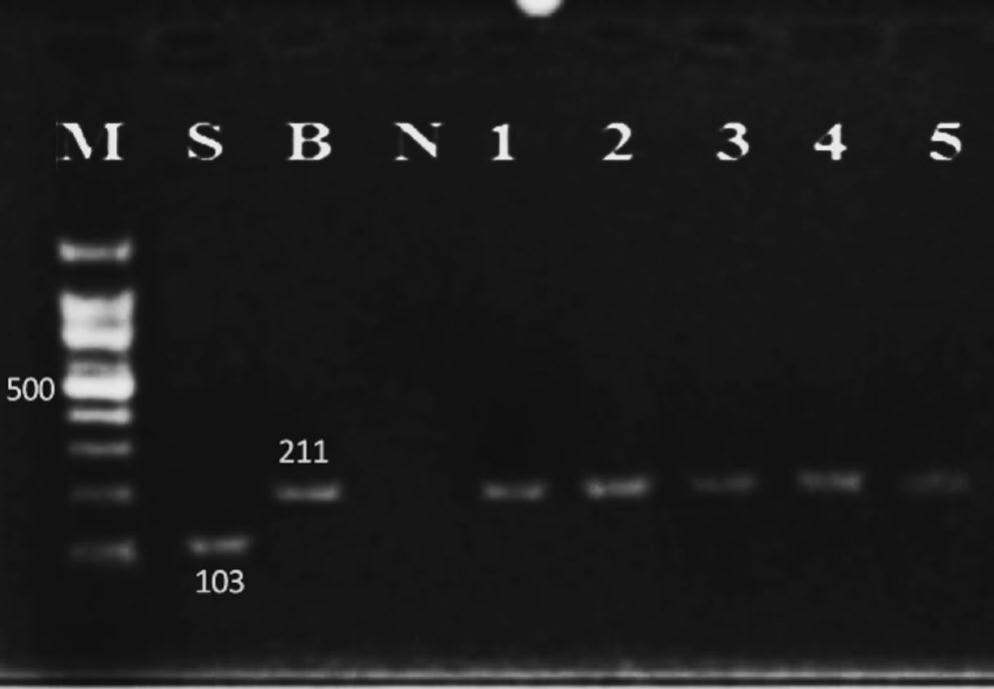
2% gel agarose electrophoresis of *Lophomonas* patient by multiplex amplification technique. M: Marker (100 bp). S: *L. striata* positive control (103 bp). B: *L. blattarum* positive control (211 bp), N: Negative control (distillated water) and 1–5: *Lophomonas* positive patients.

Out of the 120 BAL specimens tested, 30 (25%) were found to be positive. Our results revealed that out of the 30 positive specimens, 26, 29, and 30 were positive by Giemsa staining (see Figure 2), conventional PCR (see Figure 3), and multiplex‐PCR (see Figure 4), respectively, indicating a high sensitivity of these diagnostic techniques (Table 1). The sensitivity for Giemsa staining, conventional PCR, and multiplex‐PCR was found to be 86.2%, 96.6%, and 100%, respectively. Specificity was also found to be 100% (10/10) as all 10 control specimens from patients with other diseases were negative by all methods, and there were no cross‐reactions with the control specimens. Moreover, the positive likelihood ratio (LR+) for the three diagnostic tests was infinite, while the negative likelihood ratios (LR−) were 0.13, 0.03, and 0 for Giemsa staining, conventional PCR, and multiplex PCR, respectively.

**TABLE 1:** The sensitivity, specificity, positive predictive value (PPV), negative predictive value (NPV) and accuracy of different diagnostic tests for diagnosis of *Lophomonas* infection.

Method	No. of examined	Sensitivity (%) (95% CI)	Specificity (%) (95% CI)	PPV (%)	NPV (%)	Accuracy (%)
Giemsa stained smear	30	86.2 (69.4–94.5)	100 (72.2–100)	100	71.4	90
Conventional PCR	30	96.6 (82.8–99.4)	100 (67.6–100)	99.9	90.9	97.5
Multiplex‐ PCR	30	100 (88.3–100)	100 (72.2. 100)	100	100	100

**FIGURE 5:**
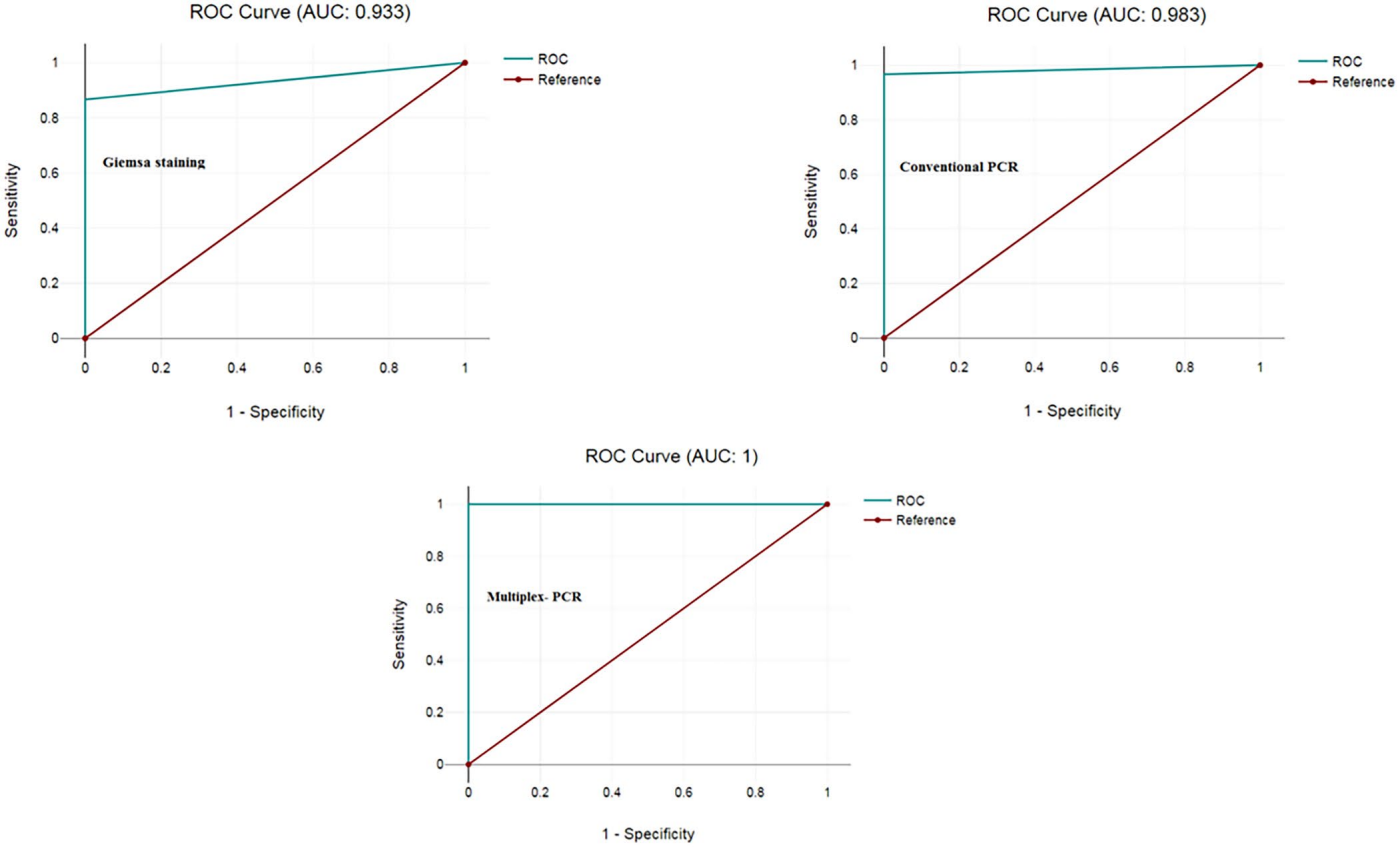
ROC curve for evaluating the sensitivity and specificity of three diagnostic tests for lophomoniasis.

The receiver operating characteristic (ROC) curve for three diagnostic tests was shown in Figure 5. The area under the curve (AUC) was 0.933, 0.983, and 1 for Giemsa staining, conventional PCR, and multiplex‐PCR, respectively.

The agreement between multiplex‐PCR and conventional PCR was found to be the highest (κ = 0.96), indicating strong agreement. However, the degree of agreement between conventional PCR and microscopy techniques was not satisfactory (κ = 0.44). The lowest agreement was observed between multiplex‐PCR and microscopy methods (κ = 0.16) (Table 1).

However, *L. striata* was not detected in any of the specimens (see Figure 4).

## 4 Discussion

Lophomoniasis poses a challenge in diagnosis due to the lack of well‐established diagnostic methods. To address this issue, we conducted a study evaluating three microscopic examinations, conventional PCR, and multiplex‐PCR using 30 bronchoscopy specimens. Our study revealed that the microscopic method (Giemsa staining) had low efficiency (85.3%) with a sensitivity of 86.2% and a specificity of 100%. The sensitivity of staining methods can be influenced by several factors, such as the type of Giemsa stain used and the quality of the BAL specimen [15]. Moreover, in some cases, due to the remarkably pleomorphic nature of the trophozoites of *Lophomonas*, they may be misdiagnosed as other atypical respiratory epithelial cells. Additionally, low parasite density may serve as a low sensitivity of Giemsa stained smear [4].

The most commonly used method for detecting the flagellated *Lophomonas* parasites is microscopic examination, particularly the wet mount smear [5, 6]. Although this method is widely available and cost‐effective, it has low sensitivity. Wet mount examination is useful in quickly assessing fresh clinical specimens and distinguishing slightly ciliated respiratory epithelial cells like creola bodies and cilicytophthoria [16] from parasites. Stained smears, on the other hand, enable comprehensive identification of the morphological characteristics of the parasite, increasing the chances of detection from suspected respiratory cells [4, 9]. Factors that contribute to the reduced sensitivity of the microscopic method include technician skill and knowledge in parasite detection, the duration dedicated to microscopic detection, and appropriate specimen collection and storage [17, 18]. Our findings suggest that changes in the staining process could improve the sensitivity of Giemsa staining, which is a rapid, inexpensive, and accessible method in small laboratories [19].

Various staining methods, including Giemsa, Papanicolaou, Trichrome stain, and Hematoxylin/Eosin, have been described in the literature for detecting *Lophomonas* [4, 20, 21, 22, 23]. However, only a few studies have evaluated the sensitivity and specificity of these staining methods. Trichrome stain has been found to provide better performance in showing the morphological characteristics of protozoa, while Giemsa and Papanicolaou staining methods have had moderate performance [16]. Additionally, there has been no success in isolating and proliferating protozoa from clinical specimens in culture media, and no serological test for this infection has been described yet [4, 5].

Overall, due to the limitations of microscopy in detecting *Lophomonas*, molecular diagnostic assays have been recommended to avoid misdiagnosis of *Lophomonas* parasites from bronchial and other respiratory cells, as a promising alternative due to their high sensitivity and specificity. Although several studies have evaluated PCR‐based methods, few have compared the performance of different molecular diagnostic assays. Therefore, further research is required to determine the optimal diagnostic method for lophomoniasis.

The present study reports the comparative performance of multiplex‐PCR and conventional PCR for detecting *Lophomonas* species in clinical specimens. Our results demonstrate that multiplex‐PCR exhibits higher sensitivity and specificity (100%, 100%) compared to conventional PCR (96.6%, 100%). In addition, the efficiency of the multiplex‐PCR assay was 100%, which is superior to other methods previously described. Importantly, all 30 specimens tested in our study were identified as *L. blattarum* using the multiplex‐PCR assay. This assay represents a valuable tool for identifying the characteristics of *Lophomonas* species and is particularly useful for detecting parasite infections in patients with atypical forms of the parasite that may be missed by microscopic examination. Briefly, the multiplex‐PCR assay described in this study represents a highly sensitive and specific tool for diagnosing lophomoniasis. It is especially valuable in situations where traditional microscopy‐based methods are limited, such as when the parasite density in clinical specimens is low.

Previous studies have reported cases of lophomoniasis co‐infected with other pathogens such as invasive pulmonary aspergillosis and COVID‐19 that were detected by conventional PCR [9, 24, 25]. A study conducted from 2017 to 2019 at the INRCL utilized both microscopic examination and SSU rRNA PCR methods to confirm *Lophomonas* infection in patients. Out of 321 specimens examined, 45 (14%) tested positive for *Lophomonas* spp. This study highlights the effectiveness of conventional PCR as a reliable tool for confirming *Lophomonas* infections in clinical samples [9].

Another retrospective analysis from 2020 to 2021 examined BAL specimens, revealing a 27.3% positivity rate for *Lophomonas* spp., using conventional PCR in Southwestern Iran. This work indicates that PCR can successfully detect this pathogen in archived BAL samples [10].

Recent studies have highlighted the occurrence of lophomoniasis among patients suspected of having tuberculosis (TB) using conventional PCR in Iran, particularly in the provinces of Mazandaran and Golestan, northern Iran. This emerging respiratory disease is caused by the protozoan *L. blattarum* and presents clinical symptoms that can closely mimic those of TB, complicating diagnosis and treatment [11, 12].

A significant study conducted in Golestan Province from 2019 to 2020 involved 216 participants who exhibited symptoms such as chronic cough, fever, and sputum production—common indicators of both lophomoniasis and TB. The study utilized PCR techniques to detect *Lophomonas* DNA in sputum samples. The results revealed that 47 patients (21.75%) were infected with *Lophomonas* spp., while 9 patients (4.2%) were diagnosed with tuberculosis. Notably, two patients were found to have co‐infections of both *Lophomonas* and TB, highlighting the potential for misdiagnosis when clinical presentations overlap [11].

In our study, we observed a high degree of agreement (κ = 0.96) between the two molecular methods employed, and we suggest that conventional PCR can be used as an alternative in situations where multiplex‐PCR is not accessible. Importantly, we found that running the three tests (multiplex‐PCR, conventional‐PCR, and microscopic examination) concurrently yields the fewest cases of misdiagnosis.

The ROC analysis and the reported AUC values indicate the following: Giemsa staining (AUC = 0.933) method demonstrates a high level of accuracy in distinguishing between positive and negative cases, suggesting it is a reliable diagnostic tool. Conventional PCR (AUC = 0.983) shows an even higher accuracy than Giemsa staining, indicating it is very effective at correctly identifying cases. Multiplex‐PCR (AUC = 1) indicates perfect accuracy in distinguishing between positive and negative cases, making it the most reliable method among the three evaluated. Overall, these AUC values highlight the strong performance of each method, with multiplex‐PCR being the most accurate for diagnostic purposes. These metrics are crucial for assessing the reliability of these techniques in clinical settings, guiding healthcare professionals in choosing appropriate diagnostic tools for detecting specific conditions or pathogens.

It is worth noting that the sensitivity of PCR can be influenced by several parameters, including the size of the primers used, the parasite species, the parasite burden, the type of gel, and the DNA extraction technique [26]. However, the PCR test has certain drawbacks, including contamination, non‐specific products, low amplification efficiency, and challenges related to annealing temperature and primer concentrations. Therefore, it is essential to optimize the PCR technique before use in each laboratory. Therefore, careful optimization of the assay is essential to obtain reliable and accurate results. The results presented in this paper provide the first report of molecular species discrimination for *Lophomonas*. This study demonstrates the suitability of variable 18S rRNA regions for the selection of *Lophomonas* species‐specific primers for two *Lophomonas* species, *L*. *blattarum* and *L. striata* . The gene‐targeting approach for the detection and confirmation of *Lophomonas* spp. has been shown to be promising with regard to high specificity and sensitivity. This rRNA multiplex‐PCR method is suitable for the diagnosis of *Lophomonas* infections in clinical human specimens at both the genus and species levels, and the 18S rRNA target can be utilized as a suitable marker for future primer design to identify this parasite. The PCR technique is advantageous because it can detect non‐cultivable pathogens before the appearance of their antigen and antibody [17], and this method promotes the detection of multi‐flagellate protozoa when culture media and serological detection methods are unavailable.

However, there are certain drawbacks to the PCR test, such as contamination, lack of multiplication or non‐specific products, low annealing, and appropriate primer concentrations [27]. The high cost of the molecular method and the lack of access to this method in all clinical laboratories make the wet mount method a routine diagnostic technique for this emerging protozoan parasite [4, 5, 21]. The number of reported cases of this neglected parasite is increasing worldwide [3], highlighting the need for trained and experienced medical personnel for prompt and subsequent detection. One of the advantages of the molecular method developed in this study is that there is room for further progress in determining drug resistance genes. The PCR approach has the potential to detect parasites in symptomatic patients using small amounts of target material, which is a significant advantage. Despite its sensitivity, many diagnostic laboratories still use microscopy for parasite detection because of its low cost and accessibility, making it a practical method for preliminary screening of *Lophomonas* infection.

The current study did not identify *L. striata* in any of the specimens, which is consistent with the absence of reported human cases of infection with this parasite. It is possible that *L. striata* is a non‐pathogenic parasite and does not cause symptomatic disease in humans. To date, *L. striata* and *L*. *blattarum* have only been isolated from cockroaches [1]. Further investigation is needed to determine the presence of this parasite in humans and to explore the potential for zoonotic transmission from animals and insects.

Future studies can utilize molecular methods on different clinical specimens to diagnose the parasite, and frozen samples can be tested in retrospective studies. The geographic distribution of *Lophomonas* in other countries and in remote areas without access to PCR diagnostic methods can also be studied using molecular methods. Additionally, further research is needed to understand the prevalence and clinical significance of *L. striata* in humans, including the potential for co‐infections with other pathogens.

Limitations: The study has several limitations that should be recognized. Firstly, the sample size was relatively small, and the data were collected from a single center. A larger sample size would enhance the robustness of the results and enable more definitive conclusions regarding the diagnostic performance of the methods used. Secondly, the study lacked a comprehensive design that included testing additional primers against other genetic markers. This limitation may affect the overall validity of the findings and their applicability to broader contexts. Thirdly, a significant limitation identified in the present study is the analytical sensitivity of the PCR methods employed. This limitation arises from the lack of access to pure *Lophomonas* parasites in clinical samples, as well as the absence of a standardized culture medium for their purification.

## 5 Conclusion

Our study underscores the superior performance of multiplex‐PCR in comparison to conventional microscopic diagnostic methods for detecting *Lophomonas* infection. The results highlight the potential of multiplex‐PCR as a reliable and powerful tool for identifying *Lophomonas* species, offering promising prospects for improved diagnostic accuracy in cases of lophomoniasis. The high degree of agreement between multiplex‐PCR and conventional PCR indicates that this approach is applicable for the accurate and valid diagnosis of *Lophomonas* infections. For this reason, it is highly recommended to perform a PCR test to confirm the results of microscopic examination and prevent potential diagnostic pitfalls. Furthermore, our results suggest that 18S rRNA may serve as an optimal molecular marker for further investigation. As a whole, further studies are needed to develop a non‐invasive, accessible, and inexpensive diagnostic method, such as a point of care test, for the diagnosis of pulmonary lophomoniasis. It is also crucial to define a standard Giemsa method for the detection of *Lophomona*s parasites, given the advantages of this method in small laboratories.

## Author Contributions

M.N., A.B., A.S., and M.F. are involved in the designing, clarification and collecting of data, and writing of the manuscript draft. M.N., S.A., H.Z.H., and M.G. were involved in editing the manuscript. M.N. and M.F. were involved in critically revising the whole manuscript. M.N. and M.F. are responsible for presenting the data and submitting the manuscript. All authors reviewed and approved the final version of the manuscript.

## Ethics Statement

This study was reviewed and approved by the research ethics committee of Mazandaran University of Medical Sciences (IR.MAZUMS. REC.1398.6871). Written informed consent was obtained from all subjects who referred to the INRCL for this study.

## Consent

Informed consent for publication of identifying information/images in an online open‐access publication was obtained from all the subjects whose identifying information is present in the manuscript.

## Conflicts of Interest

The authors declare no conflicts of interest.

## Data Availability

The data are available to the corresponding author and can be reached on request.
